# Cyclic homodimer formation by singlet oxygen-mediated oxidation of carnosine

**DOI:** 10.3389/fchem.2024.1425742

**Published:** 2024-08-19

**Authors:** Hiroko Kawakami, Yuki Itakura, Tetsuya Yamamoto, Taku Yoshiya

**Affiliations:** ^1^ Peptide Institute, Inc., Osaka, Japan; ^2^ Graduate School of Science and Engineering, Kagoshima University, Kagoshima, Japan; ^3^ Institute for Protein Research, Osaka University, Osaka, Japan

**Keywords:** carnosine, imidazole-containing dipeptides, methylene blue, photooxidation, reactive oxygen species, singlet oxygen

## Abstract

Although carnosine (β-Ala-L-His) is one of physiological protectants against *in vivo* damages caused by reactive oxygen species (ROS), its reactivity against singlet oxygen (^1^O_2_), an ROS, is still unclear at the molecular level. Theoretically, the reaction consists of two steps: i) oxygenation of the His side chain to form an electrophilic endoperoxide and ii) nucleophilic addition to the endoperoxide. In this study, the end product of ^1^O_2_-mediated carnosine oxidation was evaluated using 2D-NMR and other analytical methods both in the presence and absence of external nucleophiles. Interestingly, as the end product without external nucleophile, a cyclic homodimer was confirmed under our particular conditions. The reaction was also replicated in pork specimens.

## 1 Introduction

**FIGURE 1:**
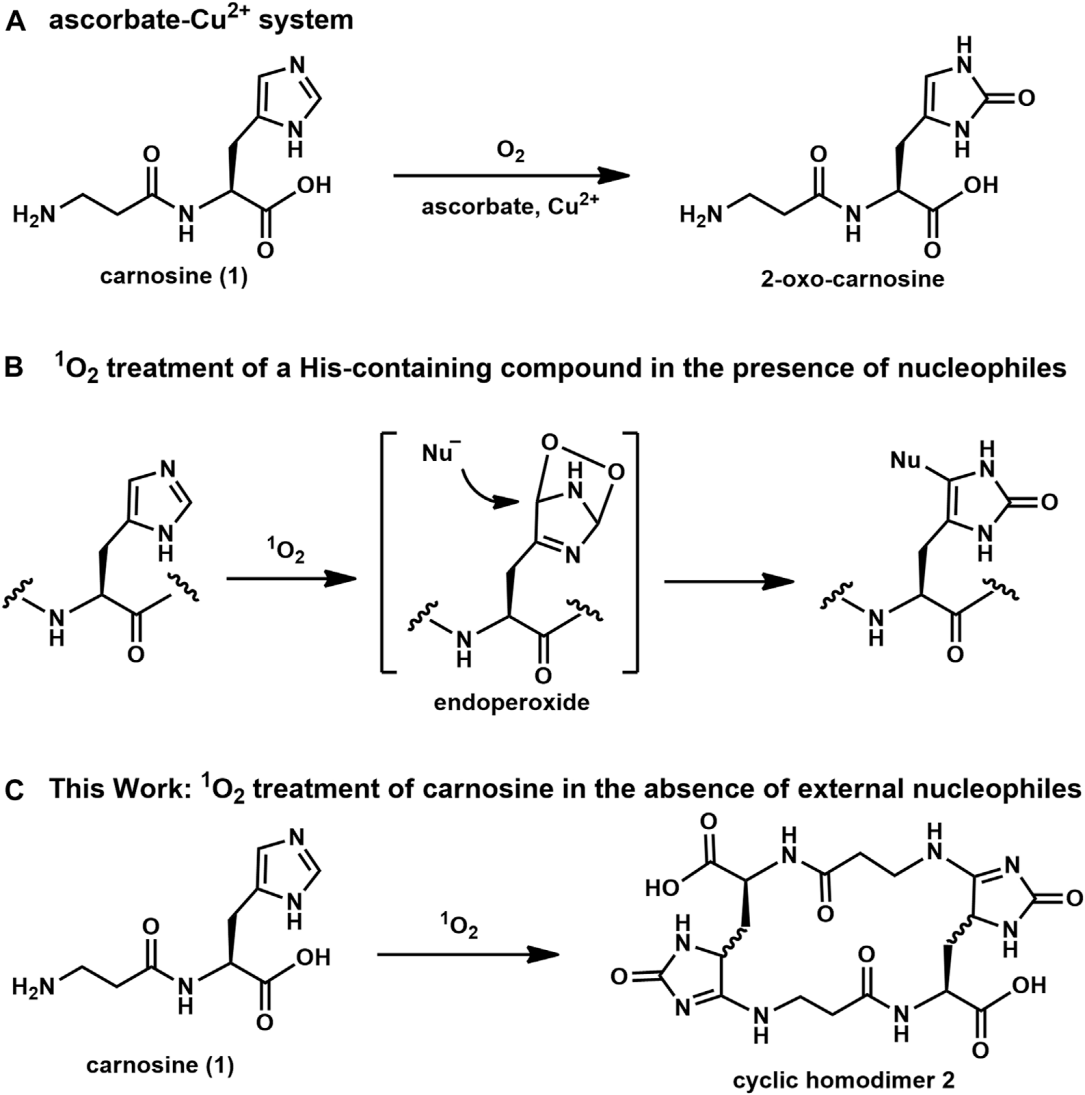
Oxidation reactions of imidazole derivatives: **(A)** ascorbic acid-copper ion system, **(B)** selective labeling of His in the presence of nucleophiles, **(C)** ^1^O_2_-mediated oxidation of carnosine (**1**) to afford cyclic homodimer **2**.

Carnosine, a dipeptide consisting of β-Ala-L-His, is known as a physiological antioxidant for mammals and is widely used as the principal compound in antioxidative supplements (Hartman et al., 1990; Ryu et al., 1997). However, their antioxidant properties against reactive oxygen species (ROS) have not been studied extensively at the molecular level. For example, in 2018, Ihara *et al.* reported that the His residue in imidazole-containing dipeptides (IDPs), such as carnosine, was converted to two-oxo-histidine by an oxidation reaction in the presence of ascorbate and Cu^2+^ (Figure 1A) (Ihara et al., 2019). These oxidative conditions appear to mimic the physiological oxidation reactions caused by hydroxyl radicals (Cheignon et al., 2016). However, the reaction of singlet oxygen (^1^O_2_), a well-known ROS, with carnosine has not been evaluated at the molecular level. Although imidazole reacts with all ROS, its reaction with ^1^O_2_ is markedly different than with hydroxyl radical. That is, the hydroxyl radical directly affords stable oxidized imidazole compounds, such as two-oxo-histidine; however, in contrast, ^1^O_2_ introduces a highly reactive temporal endoperoxide at the imidazole ring, which subsequently reacts with a nucleophile to afford a stable imidazole adduct as an end product (Figure 1B). This suggests that not only ^1^O_2_-acceptor like imidazole but also a proper nucleophilic scavenger is required to quench ^1^O_2_ reactivity; otherwise, an undesired reaction between ^1^O_2_-activated imidazole and a nucleophilic moiety in the structure of the biologically important molecule may deteriorate its normal biological activity. Therefore, we were interested in the final product of the reaction between ^1^O_2_ and carnosine. We hypothesized that carnosine, which contains both a nucleophilic N-terminal amino group and ^1^O_2_-reacting imidazole in a single molecule, could quench ^1^O_2_ by itself. We hypothesize that endoperoxide introduced by the activation of imidazole by ^1^O_2_ in carnosine can be quenched intramolecularly by the nucleophilic reaction of the N-terminal amino group. In this study, the reaction between ^1^O_2_ and carnosine in the presence of an external simple nucleophile was first evaluated using several NMR techniques to determine the local structure of imidazole adduct compounds and to clarify their NMR chemical shifts. Then, carnosine was reacted with ^1^O_2_ in the absence of external nucleophiles, and the conversion to a cyclic homodimer, not a monomeric cyclic compound, was confirmed (Figure 1C). The reproducibility of this conversion *in vivo* was estimated using pork specimens.

## 2 Results and discussion

### 2.1 Model reaction using a simple nucleophile benzylamine

**FIGURE 2:**
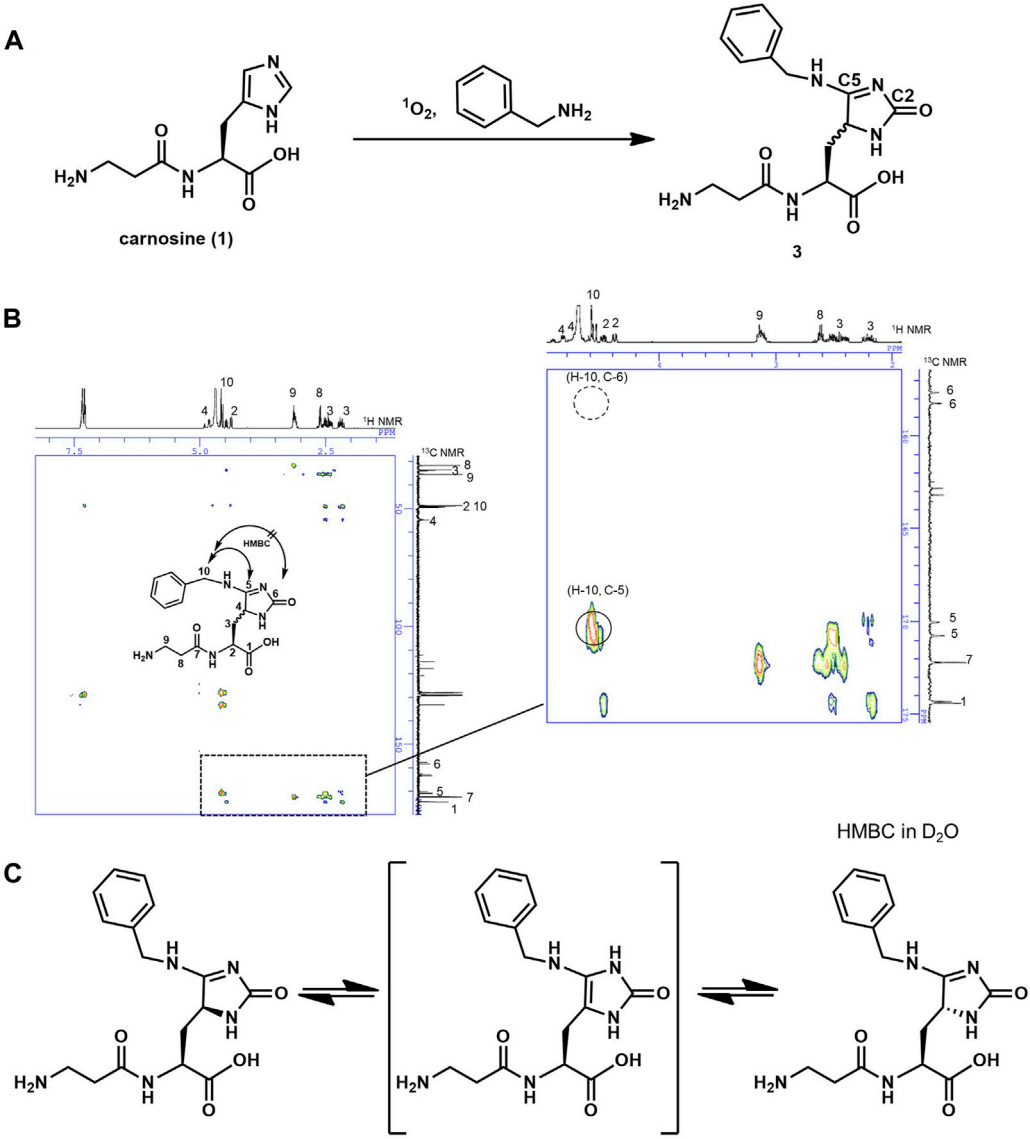
**(A)** Reaction scheme of the formation of benzylamine-adduct **3** with ^1^O_2_ in the presence of benzylamine. **(B)** HMBC spectra of compound **3** in *d*_6_-DMSO showing coupling between H-10 and C-5 (marked by circle on close-up view in the right rectangle). **(C)** The various tautomeric forms of compound **3**.

Sato group successfully applied this ^1^O_2_ system to selectively label a protein of interest at His with various nucleophiles (Nakane et al., 2021). In this method, a short-lived electrophilic endoperoxide of His is first generated by ^1^O_2_. Then, nucleophilic labeling reagent “1-methyl-4-aryl-urazole” attacks the endoperoxide, resulting in a crosslinking reaction (Figure 1B). Uesugi group also applied this ^1^O_2_ system to selective protein labeling (Toh et al., 2022). However, structural elucidation of the end products of such reactions is tricky because there are two possible electrophilic carbons, C2 and C5, in the endoperoxide resulting from the [4 + 2] cycloaddition between ^1^O_2_ and imidazole. So far, such a structure with C5 adduct has been reported when the nucleophile is aromatic nitrogen (Shen et al., 2000; Nakane et al., 2021). Thus, we first attempted to characterize the end product using benzylamine as a structurally simple small aliphatic nucleophile to estimate the end products of the ^1^O_2_–carnosine reaction. In the presence of methylene blue (MB) as a photosensitizer, carnosine was oxygenated by ^1^O_2_ under LED irradiation at 660 nm (so-called “photooxygenation”) in the presence of excess benzylamine (Figure 2A). Carnosine-benzylamine adduct **3** thus obtained was isolated by HPLC purification, and its structure was determined using several analytical methods, such as ESI-MS, ^1^H, and ^13^C NMR with the aid of 2D-NMR measurements. Notably, as shown in Figure 2B, our HMBC experiments confirmed a correlation between the benzyl-positioned proton (H10) and the C5 carbon but not the C2 carbon; while, MG-H1, which contains the characteristic structure of the C2-adduct product, was recently synthesized chemically, and the HMBC correlation at C2 was confirmed (Supplementary Figure S1) (Wang et al., 2012). These results indicate that a nucleophilic attack occurred at the C5 position. This is probably due to electron density distribution of the electrophilic endoperoxide intermediate. Additionally, in our ^1^H NMR experiment, a proton at the C4 position was first observed; however, it disappeared over time in D_2_O, suggesting a D/H exchange (Supplementary Figure S2). This result indicates that **3** has a tautomer around the imidazole ring (Figure 2C) to racemize the C4 position. Both ^1^H and ^13^C NMR spectra showed separate peaks corresponding C4. Based on these results, we concluded that carnosine was converted to the benzylamine adduct **three** by ^1^O_2_ in the presence of excess benzylamine.

### 2.2 Carnosine oxidation

**FIGURE 3:**
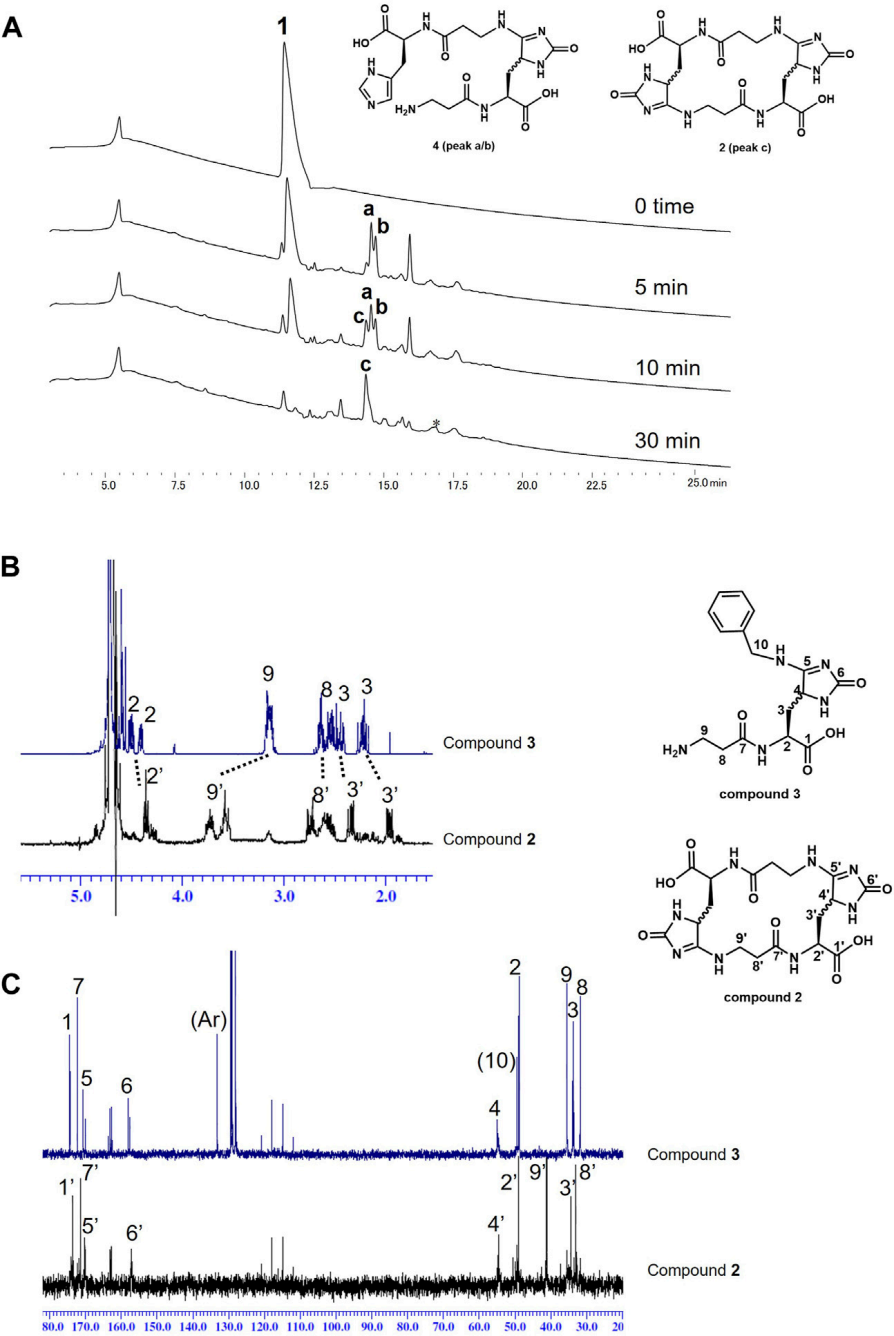
**(A)** HPLC traces of photo-oxidation reaction of carnosine (**1**). *Trace of cyclic trimers were eluted in this broad peak. (B/C) NMR spectra comparison of compounds **3** and **2**: **(B)** ^1^H and **(C)** ^13^C. Differences at H-9 and C-9 were mainly observed in both spectra.

Next, carnosine was photooxygenated using MB and LED light (660 nm) in the absence of a nucleophile to evaluate whether carnosine could quench ^1^O_2_ by itself (Figure 3). Similar to the above experiments, carnosine was photooxygenated in the absence of benzylamine. The reaction progress over time was analyzed by HPLC (Figure 3A). The consumption of carnosine (**1**) was observed at an early stage, and the product was a complex mixture at that time; however, after 30 min, peak **c** showing mass number m/z 481.2 [M + H^+^] was mainly observed. Thus, peak **c** was isolated by HPLC and characterized using the above-mentioned methods including ^1^H and ^13^C NMR. The NMR spectra of the compound from peak **c** were very similar to those of compound **3** except for the carbon and proton at C9 (Figure 3B). D/H exchange was observed with time for the proton at C4 (Supplementary Figure S4). These results suggest that the compound from peak **c** has an aligned circular structure, namely, cyclic homodimer **2**. Additionally, peaks **a** and **b** with mass numbers of m/z 468.2 [M + H^+^] were transiently observed before the formation of cyclic homodimer **2** at five or 10 min. Based on the structure of cyclic homodimer **2** as the end product, the compounds eluted at peaks **a** and **b** were estimated to be intermediate linear dimers **4**. Interestingly, because no polymers other than trace amounts of cyclic trimers were significantly observed, cyclic dimers seem to be the preferred product in this reaction.

### 2.3 Biological reproducibility

**FIGURE 4:**
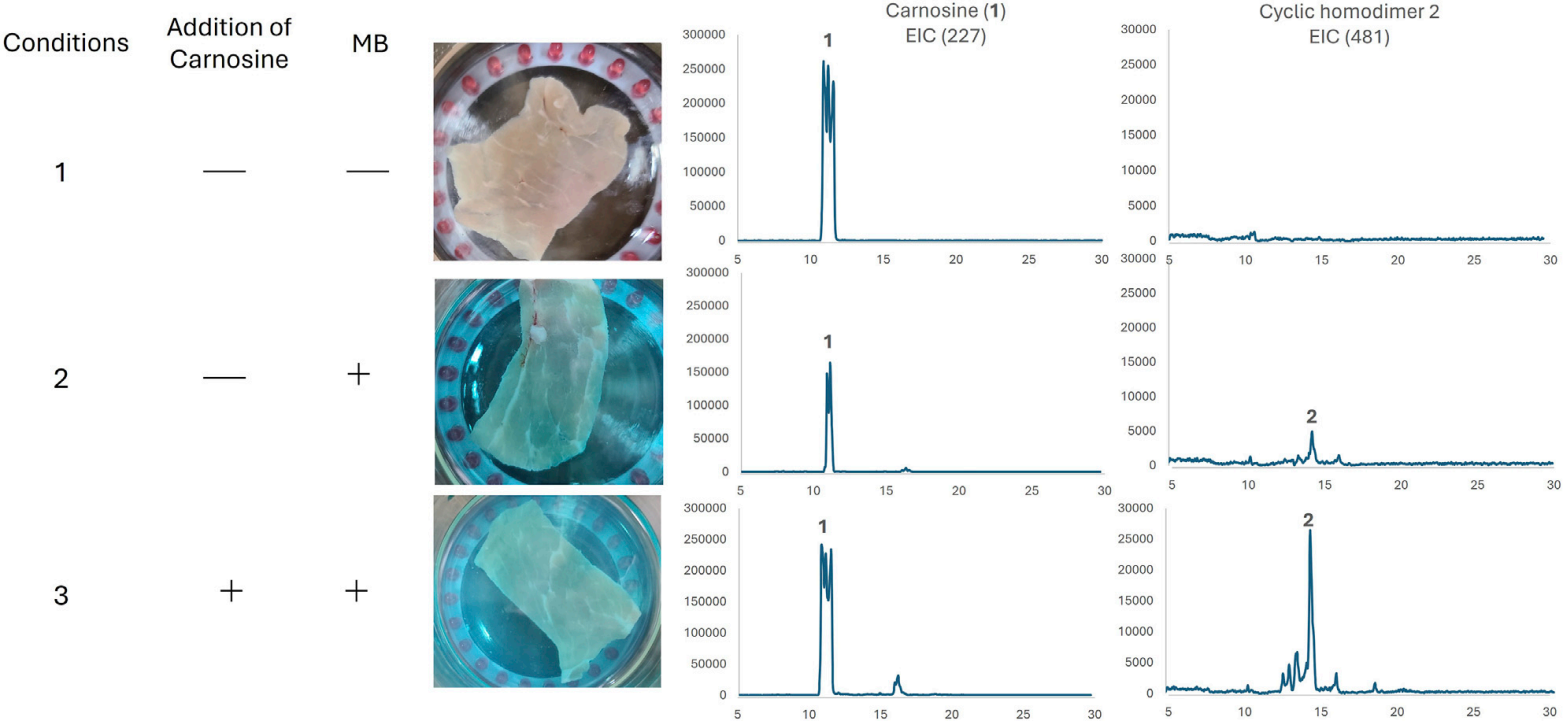
Photo-oxidation of a piece of pork. The reaction was monitored by LC-MS and extraction chromatography (EIC) was used for product evaluation.

Various compounds other than carnosine exist in natural environments. Thus, to evaluate whether this transformation could occur *in vivo*, we used a piece of pork as a biological sample for photo-oxygenation (Figure 4) because pigs are known to contain high levels of carnosine (Kondo, 2005). Pieces of pork were exposed to 660 nm red light for 40 min in the absence or presence of the photosensitizer MB (Conditions one and 2, respectively). As a positive control, carnosine was added externally to a piece of pork (Condition 3). Each solution was filtered and analyzed by LC-MS. In the absence of the photosensitizer MB or the external addition of carnosine, a reasonable amount of inherent carnosine was observed. Surprisingly, in the presence of the photosensitizer MB, the formation of cyclic homodimer **2** was confirmed without the addition of external carnosine. These results suggest that animals store carnosine to protect themselves from ^1^O_2_ *in vivo* and that some carnosine may protect the body by acting as sacrificial agents by forming an oxidized homocyclic dimer.

## 3 Conclusion

We evaluated ^1^O_2_-mediated oxygenation and the subsequent carnosine reaction at the molecular level. First, using the model nucleophile benzylamine, nucleophilic addition was confirmed at the C5 position of the endoperoxide after the oxygenation of the His side chain by ^1^O_2_. Additionally, based on the results of the structural analysis of benzylamine adduct **3**, we found that cyclic homodimer **2** is one of the end products after the photooxygenation of carnosine for the first time. Because this transformation occurred even in pork specimens, carnosine may protect the body from ^1^O_2_ by sacrificing itself and forming an oxidized homocyclic dimer *in vivo*. These results may help us understand the function of carnosine in the future.

## 4 Experimental

### 4.1 General information

All reagents and solvents were obtained from the Peptide Institute, Inc. (Osaka, Japan), FUJIFILM Wako Pure Chemical Corporation (Osaka, Japan), Tokyo Chemical Industry Co., Ltd. (Tokyo, Japan), Nacalai Tesque, Inc. (Kyoto, Japan), Watanabe Chemical Industries, Ltd. (Hiroshima, Japan), and Merck KGaA (Darmstadt, Germany). Preparative HPLC was carried out on a Shimadzu liquid chromatograph Model LC-20A (Kyoto, Japan) with a TSKgel Amide-80 (21.5 × 250 mm) and the following solvent systems: 0.1% TFA in H_2_O and 0.1% TFA in CH_3_CN at a flow rate of 8 mL min^−1^ with detection at 220 nm. Analytical HPLC was performed on a Shimadzu liquid chromatograph Model LC-20A (Kyoto, Japan) with a TSKgel Amide-80 (4.6 × 150 mm) and the following solvent systems: 0.1% TFA in H_2_O and 0.1% TFA in CH_3_CN at a flow rate of 1 mL min^−1^ (40°C) with a linear gradient of CH_3_CN (90%–60% CH_3_CN, 25 min). Purities were determined based on the percentage area of the peaks detected at 220 nm. Mass spectra were obtained using an Agilent G6135B LC/MSD detector and an Agilent 1,260 Infinity II series HPLC system. ^1^H and ^13^C NMR spectra were recorded on a JEOL-ECX400 spectrometer (Tokyo, Japan) in deuterated solvents, with the solvent residual peak of DMSO-*d*_6_ (^1^H = 2.50 ppm, ^13^C = 39.53 ppm) or TMS (^1^H = 0.00 ppm) as an internal reference, unless otherwise stated.

### 4.2 Synthesis of cyclic homodimer 2

Carnosine (45.2 mg, 0.2 mmol, 1.0 equiv.) and MB (0.13 mg, 0.4 μmol, 0.002 equiv.) were dissolved in CH_3_CN/pH 8.5 phosphate buffer (1/1, 20 mL), and the solution was exposed to 660 nm red light and stirred for 50 min at room temperature. Then, the reaction mixture was diluted with CH_3_CN, and the crude solution was purified by HPLC (90%–60% CH_3_CN/H_2_O for 80 min) to obtain the title compound (8.2 mg, 0.017 mmol, 17% yield) as a lyophilized amorphous powder.

Purity (HPLC): 97.0%; ^1^H NMR (400 MHz, D_2_O): δ 4.95 (dd, 1H, *J* = 5.49 Hz, *J* = 6.40 Hz), 4.46 (dd, 1H, *J* = 7.32 Hz, *J* = 7.32 Hz), 3.87–3.64 (m, 2H), 2.87–2.60 (m, 2H), 2.42 (dd, 1H, *J* = 7.77 Hz, *J* = 14.18 Hz), 2.06 (dd, 1H, *J* = 7.32 Hz, *J* = 13.37 Hz); ^13^C NMR (100 MHz, D_2_O): δ 173.6, 171.4, 170.1, 157.2, 54.6, 49.0, 41.1, 34.4, 33.1 ppm; ESI-MS: calcd. For C_18_H_24_N_8_O_8_ [M + H^+^]:481.2, found:481.2.

### 4.3 Synthesis of benzylamine-adduct 3

Carnosine (45.2 mg, 0.2 mmol, 1.0 equiv.), benzylamine (150.0 mg, 1.4 mmol, 7.0 equiv.), and MB (0.13 mg, 0.4 μmol, 0.002 equiv.) were dissolved in CH_3_CN/H_2_O (1/1, 20 mL), and the solution was exposed to 660 nm red light and stirred for 50 min at room temperature. Then, the reaction mixture was diluted with CH_3_CN, and the crude solution was purified by HPLC (90%**–**60% CH_3_CN/H_2_O for 80 min) to obtain the title compound (25 mg, 0.072 mmol, 36% yield) as a lyophilized amorphous powder.

Purity (HPLC): 98.0%; ^1^H NMR (400 MHz, D_2_O): δ7.47-7.35 (m, 5H), 5.06 (dd, 0.5H, *J* = 5.49 Hz, *J* = 5.49 Hz), 4.91 (dd, 0.5H, *J* = 3.66 Hz, *J* = 7.78 Hz), 4.70–4.61 (m, 2H), 4.57 (dd, 0.5H, *J* = 6.40 Hz, *J* = 9.15 Hz), 4.48 (dd, 0.5H, *J* = 3.66 Hz, *J* = 10.52 Hz), 3.28–3.13 (m, 2H), 2.77–2.47 (m, 3H), 2.34–2.23 (m, 1H) ppm; ^13^C NMR (100 MHz, D_2_O): δ 174.3, 172.3, 170.7/170.0, 158.1/157.5, 133.2, 129.3, 129.0, 128.1, 54.9, 49.4, 48.9/48.7, 35.5, 33.8/33.7, 31.7 ppm; ESI-MS: calcd. For C_16_H_21_N_5_O_4_ [M + H^+^]: 348.2, found: 348.1. This compound was obtained as a diastereomixture, and NMR signals were partly separated (underlined).

### 4.4 Photooxygenation reaction using pork as a biological sample

A piece of pork (10 g) was soaked in CH_3_CN/pH 8.5 phosphate buffer (1/1, 20 mL) in the absence/presence of 0.07 mg of MB for condition 1/2, as shown in Figure 4. In the positive control experiment (Condition three in Figure 4), 25 mg of carnosine was added. Each mixture was then irradiated with an LED (660 nm) for 40 min at room temperature and analyzed using LC-MS. Because phosphate buffered reaction mixtures were directly injected into the TFA-acidified LC-MS system for carnosine analysis, carnosine was not eluted as a single peak due to lack of uniformity of salt.

## Data Availability

The original contributions presented in the study are included in the article/Supplementary Material further inquiries can be directed to the corresponding author.
