# Supplementary material for: Cyclic homodimer formation by singlet oxygen-mediated oxidation of carnosine
Source: Front Chem. 2024 Aug 19;12:1425742. doi: 10.3389/fchem.2024.1425742 (PMC11367420; doi:10.3389/fchem.2024.1425742)
Supplement: Supplementary file 1 [file DataSheet1.PDF]

Supplementary Material

Cyclic homodimer formation by singlet oxygen-mediated oxidation of carnosine

Hiroko Kawakami[1,2], Yuki Itakura[1], Tetsuya Yamamoto[1], Taku Yoshiya[1,3]

[1]Peptide Institute, Inc., 7-2-9 Saito-Asagi, Ibaraki-shi, Osaka 567-0085, Japan.

[2]Graduate School of Science and Engineering, Kagoshima University, 1-21-35 Korimoto, Kagoshima-shi, Kagoshima 890-0065, Japan

[3]Institute for Protein Research, Osaka University, 3-2 Yamadaoka, Suita-shi, Osaka 565-0871, Japan

*** Correspondence:**
Taku Yoshiya
t.yoshiya@peptide.co.jp

Contents

- **Supplementary Figures**
- **Characterization data for compound 2**
- **Characterization data for compound 3**

1 Supplementary Figures

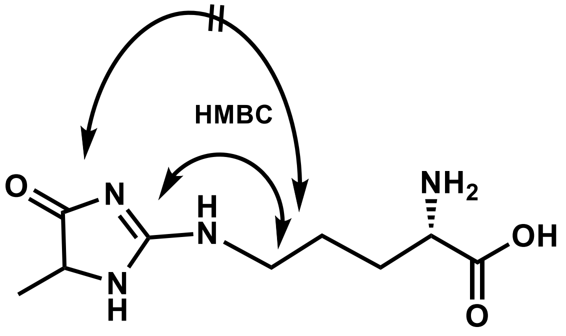

MG-H1

Supplementary Figure 1. Structure of MG-H1 and reported HMBC correlation.

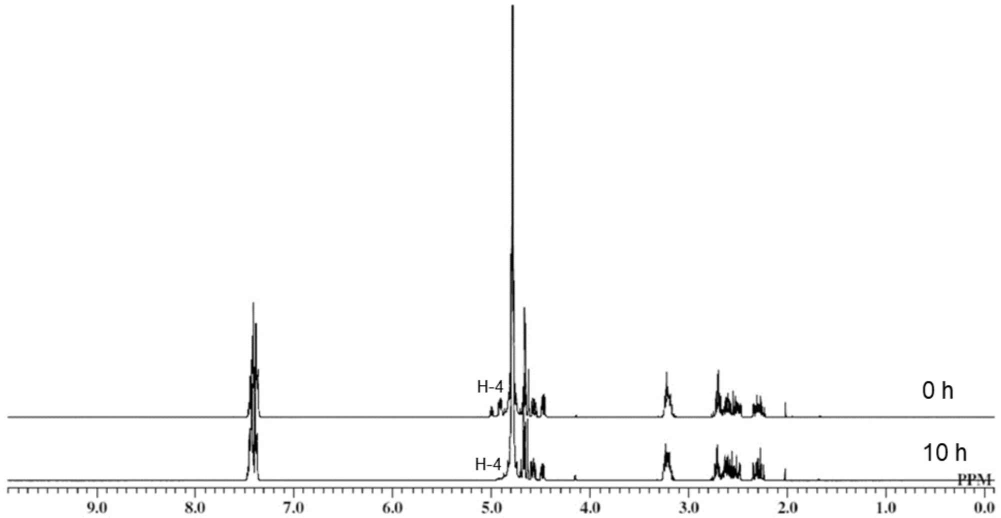

Supplementary Figure 2. Disappearance of 4-H in ^{1}H NMR spectra of compound **3** in D_2O at room temperature after 10 h. There is no significant difference between 0 h and 10 h except for the disappearance of the signal of 4-H, which was assigned with the aid of the HMBC spectrum shown in Fig. S3.

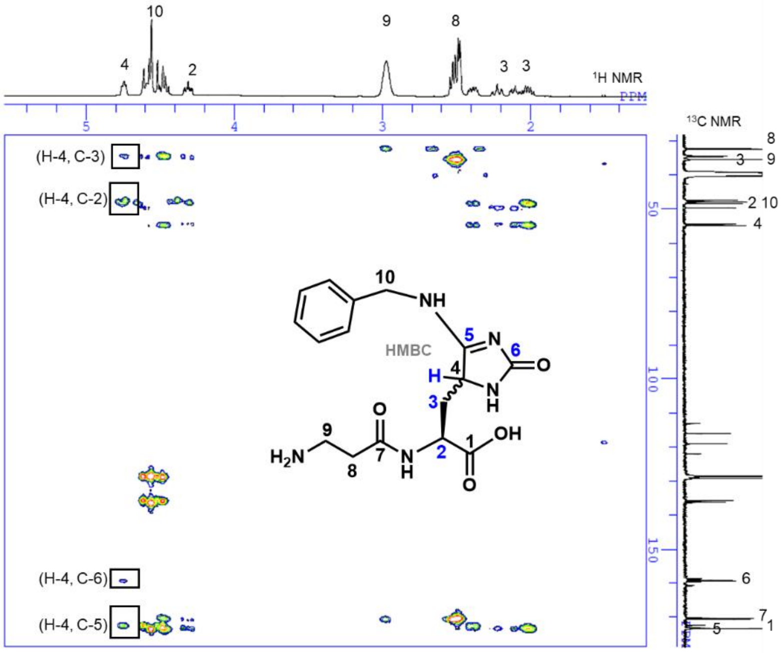

Supplementary Figure 3. The HMBC spectrum of compound **3** in d_6-DMSO shows cross peaks (marked with rectangles) between H-4 and C-2/3/5/6 (indicated with blue color).

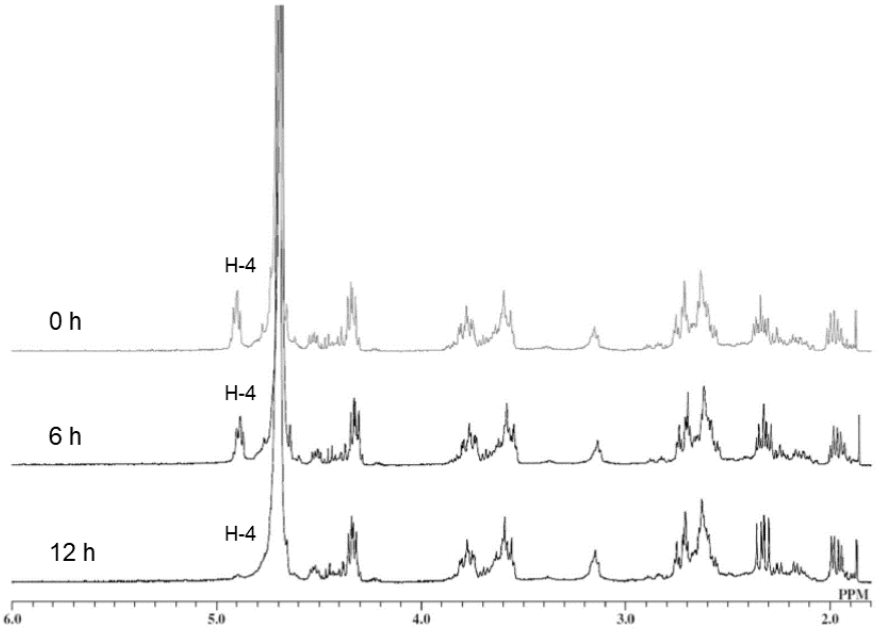

Supplementary Figure 4. Disappearance of 4-H in ^{1}H NMR spectra of compound **2** in D_2O at room temperature over 12 h. There is no significant difference over time except for the disappearance of the signal of 4-H.

2 Characterization data for compound 2

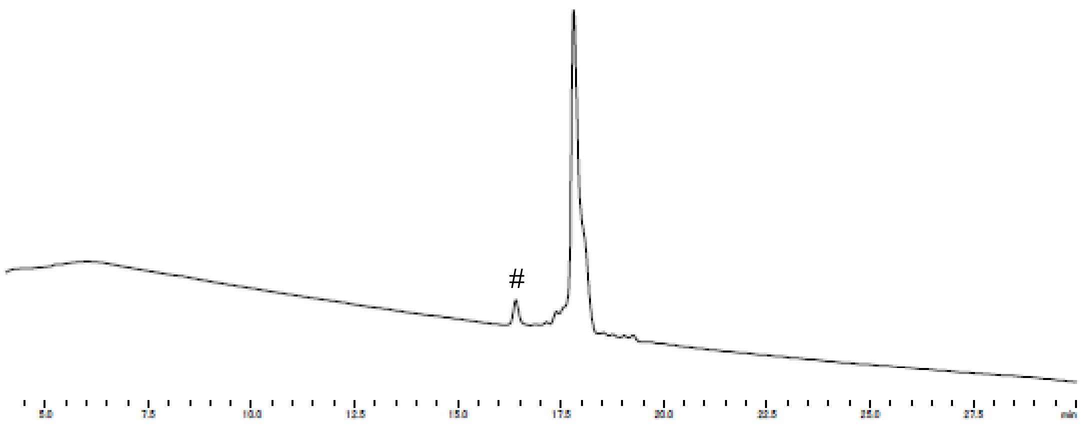

HPLC analysis of compound **2**. Peak # was confirmed to be the tautomer of **2**. Column: TSKgel Amide-80, 3um, 4.6x150 mm, Eluent: 90-60% CH_3CN/0.1% TFA (25 min), Temp:40 °C

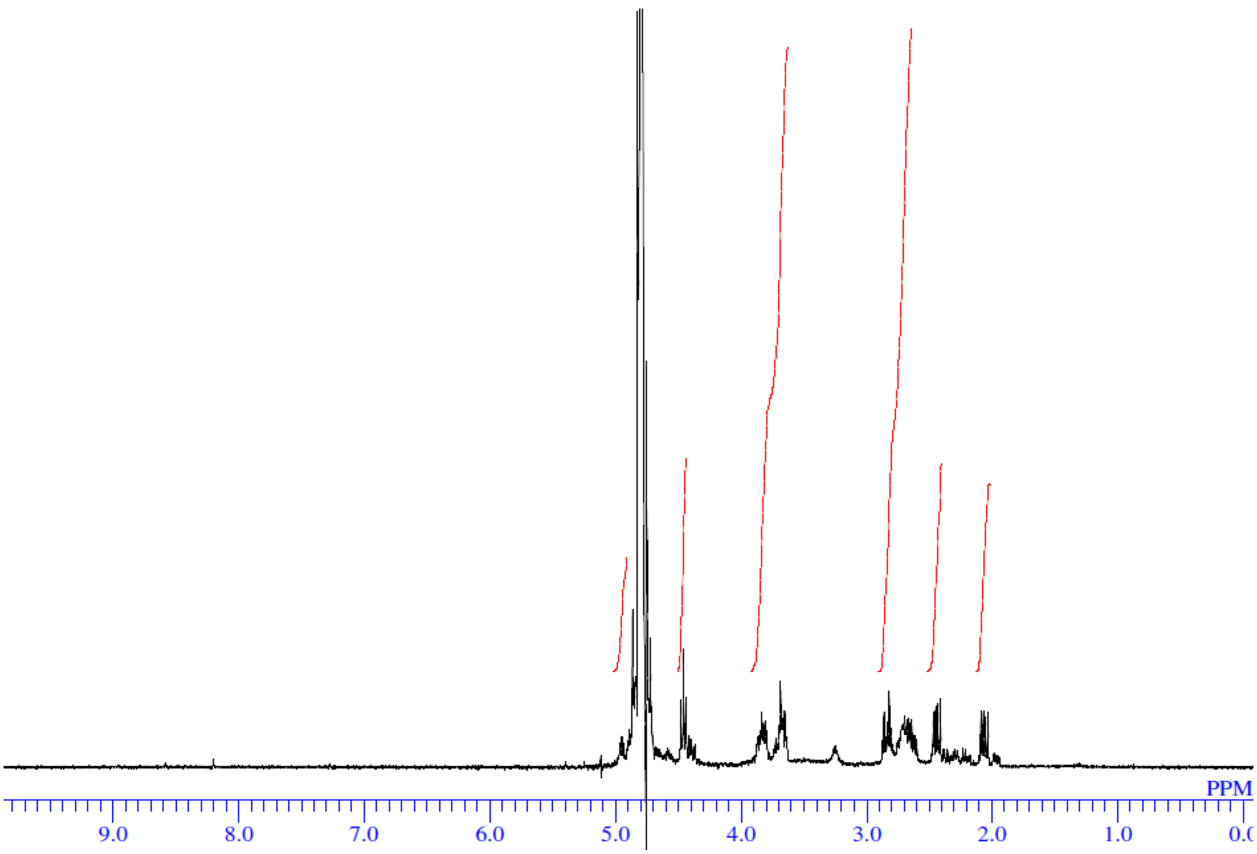

^{1}H NMR spectrum of compound **2**.

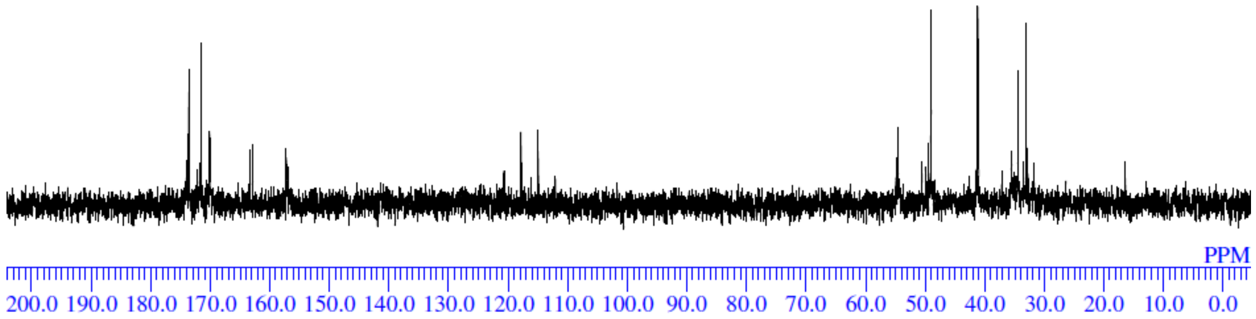

^{13}C NMR spectrum of compound **2**.

3 Characterization data for compound 3

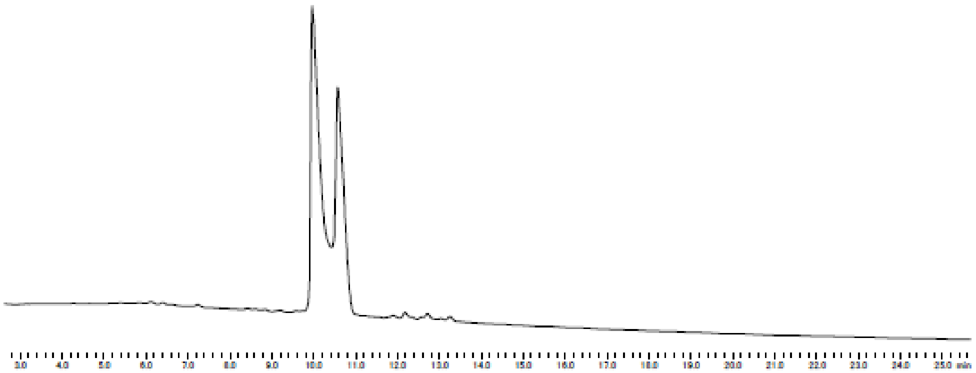

The HPLC analysis of compound **3** showed two peaks, probably due to the diastereoisomers. Column: TSKgel Amide-80, 3um, 4.6x150 mm, Eluent: 90-60% CH_3CN/0.1% TFA (25 min), Temp: 40 °C

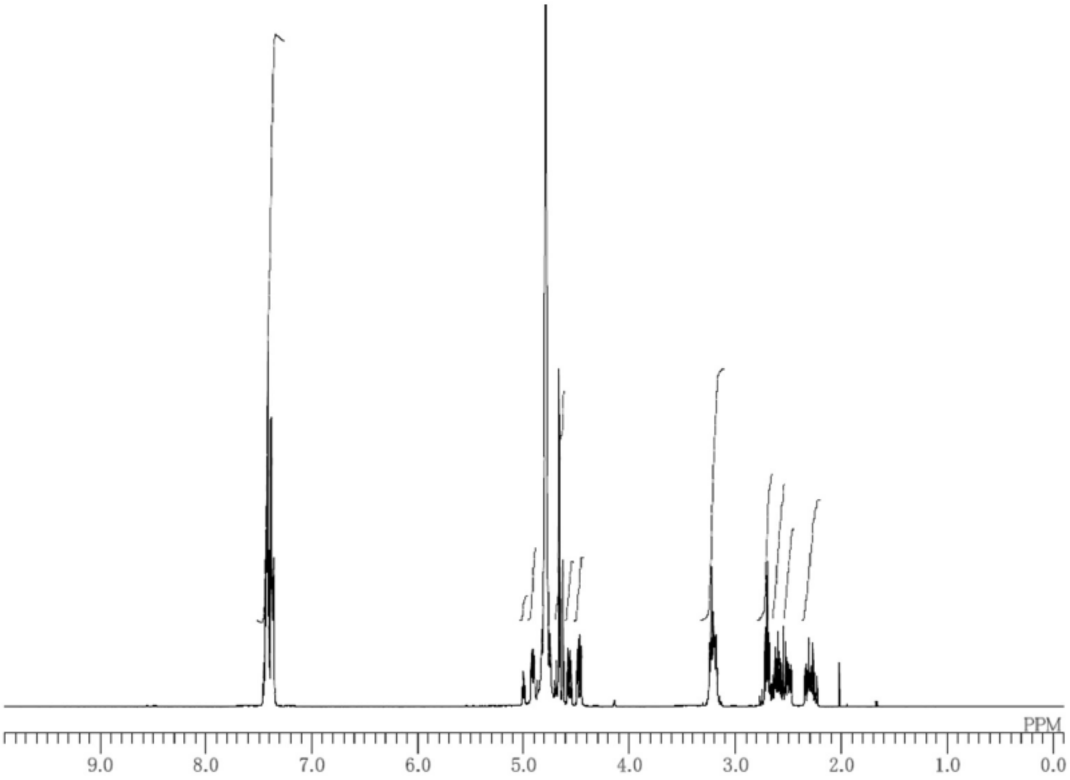

^{1}H NMR spectrum of compound **3**.

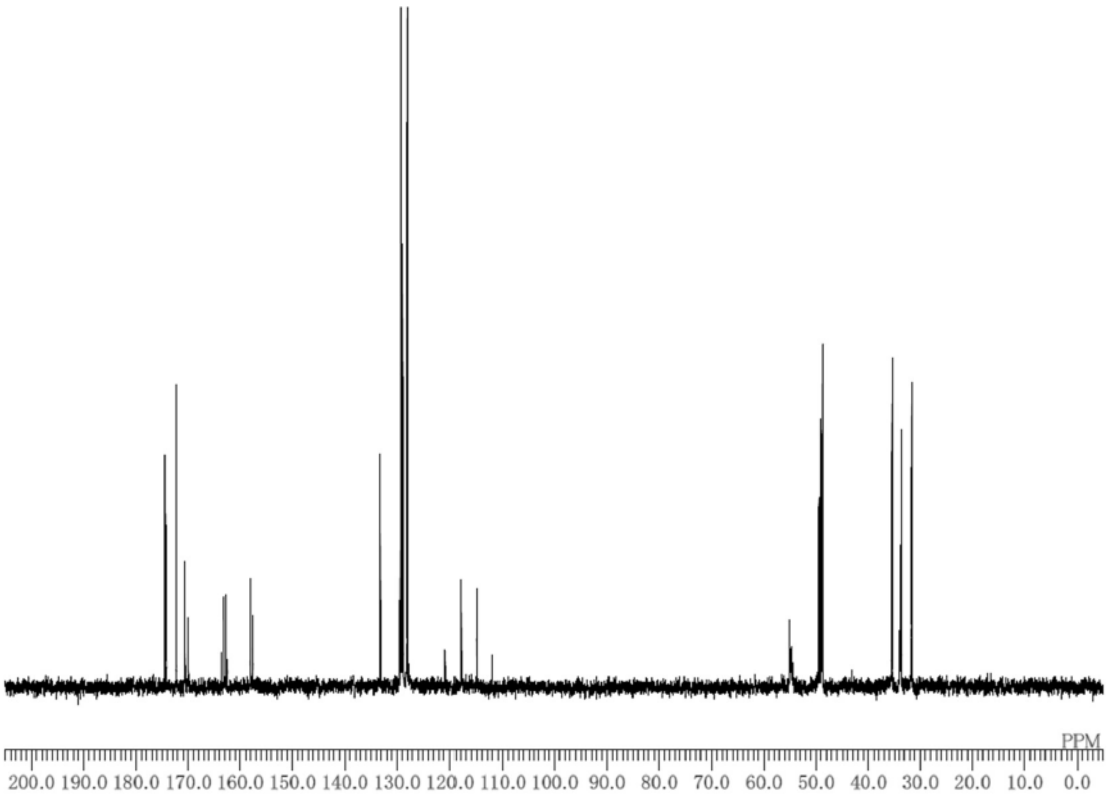

^{13}C NMR spectrum of compound **3**.
